# Identification of glycolysis- and myocardial fibrosis-related diagnostic biomarkers in heart failure: an integrative bioinformatics and machine learning study

**DOI:** 10.3389/fcvm.2026.1912375

**Published:** 2026-09-22

**Authors:** Lin Yang, Bing Liu, Shuang Qiu, Yimeng Zhang, Na Ta, Yanxia Liu

**Affiliations:** State Key Laboratory of Frigid Zone Cardiovascular Disease, Cardiovascular Research Institute and Department of General Medicine, General Hospital of Northern Theater Command, Shenyang, Liaoning, China

**Keywords:** bioinformatics, diagnostic biomarkers, glycolysis, heart failure, immune infiltration, machine learning, myocardial fibrosis

## Abstract

**Background:**

Heart failure (HF) is a leading cause of global morbidity and mortality, yet the crosstalk between energy metabolism reprogramming and myocardial fibrosis remains poorly understood. This study aimed to identify glycolysis- and myocardial fibrosis-related diagnostic biomarkers in HF through integrative bioinformatics analysis.

**Methods:**

Datasets GSE57338 and GSE76701 were obtained from the Gene Expression Omnibus database. Glycolysis-related genes and myocardial fibrosis-related genes were sourced from GeneCards and PubMed. Differentially expressed genes were identified, intersected with gene sets, and screened using three machine learning algorithms. A logistic regression diagnostic model was constructed and validated. Standard bioinformatics methods were used for functional enrichment analysis, construction of regulatory networks, and immune infiltration analysis.

**Results:**

A total of 14 glycolysis- and myocardial fibrosis-related differentially expressed genes were identified, with five key genes (*SERPINA3, HBB, NPPA, LUM*, and *OGN*) screened via machine learning. The diagnostic model achieved excellent performance in both the training (AUC=0.983) and validation (AUC=0.938) sets. Among individual genes, SERPINA3 demonstrated the highest diagnostic accuracy (AUC=0.998), while the combined model provided complementary advantages in terms of stability and multidimensional biomarker coverage. Enrichment analysis revealed involvement in blood pressure regulation and extracellular matrix organization. Immune infiltration analysis identified 11 immune cell types with differential infiltration, and regulatory networks were constructed including the potential therapeutic compounds identified.

**Conclusions:**

In this study, we identified five key genes associated with glycolysis and myocardial fibrosis in HF and constructed a robust diagnostic model with excellent predictive performance. These findings provide novel insights into the molecular mechanisms of HF and offer promising candidates for diagnostic biomarker development and therapeutic targeting.

## 1 Introduction

Heart failure (HF) affects over 60 million people worldwide and exhibits a 5-year mortality rate of approximately 50%–60%, which exceeds that of many common malignancies (1, 2). Current diagnostic approaches relying on echocardiography and natriuretic peptides are insufficient for early and precise diagnosis due to the high heterogeneity of HF (3). Therefore, elucidating the molecular mechanisms underlying HF and identifying reliable biomarkers are of critical clinical importance.

Myocardial metabolic reprogramming toward glycolysis and excessive myocardial fibrosis are two core drivers of HF progression (4, 5). Sustained neurohormonal activation induces fibroblast activation and extracellular matrix deposition, leading to myocardial fibrosis-induced pump failure (6, 7). Although the individual roles of glycolysis and fibrosis in HF have been studied, their potential crosstalk remains underexplored. Thus, integrative analysis of glycolysis-related genes (GRGs) and myocardial fibrosis-related genes (MFRGs) may identify key molecular nodes and novel biomarkers for HF.

In this study, we integrated publicly available transcriptomic datasets to screen for both GRGs and MFRGs in HF. Using a multi-algorithm bioinformatics strategy, we identified key genes, investigated their biological functions, immune infiltration characteristics, and regulatory networks, and constructed a diagnostic model.

## 2 Materials and methods

### 2.1 Datasets and data preprocessing

The gene expression datasets GSE57338 (8) and GSE76701 (9) were downloaded from the Gene Expression Omnibus (GEO) database (10) using GEOquery (11) (Version 2.70.0). GSE57338 contained 177 samples from HF patients and 136 control samples, whereas GSE76701 contained 4 samples from HF patients and 4 control samples. All samples were from human left ventricular tissue. Data were normalized using normalizeBetweenArrays (limma) (12) (Version 3.62.1).

GRGs and MFRGs were obtained from the GeneCards database (13) (relevance score > 2, protein coding) and PubMed literature (14–20). After merging and deduplication, a total of 1791 GRGs and 3339 MFRGs were identified. The intersection of these two gene sets yielded 1729 glycolysis- and myocardial fibrosis-related genes (GMFRGs). In this study, GeneCards and PubMed were used for the initial gene screening stage to adopt a broad-coverage strategy that captures as many candidate genes as possible. Curated pathway databases (KEGG, MSigDB, and Reactome) were intentionally reserved as functional validation tools in downstream analyses (GSEA/GSVA using MSigDB c2 gene sets in Section 2.3, and KEGG enrichment in Section 2.6), rather than being used for gene preselection. This design choice was made to avoid introducing pathway-restricted bias and to ensure that the subsequent differential expression and machine learning analyses remained independent of predefined pathway membership. The workflow chart is shown in Supplementary Figure S1.

### 2.2 Identification of differentially expressed genes

Differentially expressed genes (DEGs) between HF and control groups in GSE57338 were identified using limma (|logFC| > 1.0 and p < 0.05). Volcano plots were generated via ggplot2. The intersection of DEGs with GMFRGs yielded glycolysis- and myocardial fibrosis-related differentially expressed genes (GMFRDEGs), which were visualized using a Venn diagram and heatmap (pheatmap).

### 2.3 Enrichment analysis

Gene set enrichment analysis (GSEA) and gene set variation analysis (GSVA) were performed using the clusterProfiler and GSVA packages, respectively (21–23). The c2.all.v2024.1.Hs.symbols gene set (seed=2022, minGSSize=10, maxGSSize=500) and the c2.cp.v2023.2.Hs.symbols gene set were used for GSEA and GSVA, respectively. Significance thresholds were set as follows: p < 0.05 and FDR < 0.05 [Benjamini–Hochberg (BH) correction] for GSEA; p < 0.05 and top 10 positive/negative pathways by logFC for GSVA.

### 2.4 Machine learning and key gene identification

Three machine learning algorithms were applied sequentially. First, logistic regression was used to screen the 14 glycolysis- and myocardial fibrosis-related differentially expressed genes (GMFRDEGs); all 14 genes met the significance threshold (p < 0.05) and were retained for subsequent analysis. Second, these 14 genes were input into the support vector machine recursive feature elimination (SVM-RFE) algorithm (24) with 5-fold cross-validation to determine the optimal feature subset based on the highest accuracy and lowest error rate, which identified a 5-gene set (SERPINA3, HBB, NPPA, LUM, and OGN). Third, LASSO regression (25) (seed=2024, family=“binomial”) was applied to the 5 genes selected by SVM-RFE for confirmation, and all five genes were retained in the LASSO model.

### 2.5 Diagnostic model construction and validation

A logistic regression model was constructed based on the key genes identified. The risk score was calculated using the following equation:

$$RiskScore=\;\underset{i}{\sum }\;Coefficient(gene_{i})*mRNA\;Expression(gene_{i})$$

A logistic regression model was chosen for the final diagnostic model because, after the two-step feature reduction (SVM-RFE followed by LASSO confirmation), the number of predictors was reduced to a low-dimensional set of 5 genes. In this setting (5 features, 313 samples), logistic regression does not suffer from overfitting or the curse of dimensionality. Moreover, logistic regression directly outputs odds ratios (ORs) with 95% confidence intervals for each gene, providing quantifiable effect directions and magnitudes that are immediately interpretable for biomarker development—an advantage not readily available from tree-based or ensemble methods such as Random Forest or XGBoost. This interpretability is essential for potential clinical translation of the diagnostic model.

Model performance was evaluated using receiver operating characteristic (ROC) curves via the pROC package (Version 1.18.5). Calibration curves and decision curve analysis (DCA) were performed using the rms (Version 6.7-1) and ggDCA packages (Version 1.1), respectively. Functional similarity among key genes was assessed using the GOSemSim package (Version 1.18.5). Model validation was performed using the independent GSE76701 dataset. The specific intercept, regression coefficients, standard errors, odds ratios, and 95% confidence intervals for the final logistic regression model are provided in Supplementary Table S13.

We acknowledge that logistic regression may not be absolutely optimal in predictive accuracy compared with ensemble methods such as RF or XGBoost; however, its superiority in clinical interpretability makes it a more suitable choice for diagnostic model development. Future comparisons of multiple algorithms in larger cohorts would be a valuable direction.

### 2.6 Go and kegg enrichment analysis

Gene Ontology (GO) (26) and Kyoto Encyclopedia of Genes and Genomes (KEGG) (27) enrichment analyses for the key genes were conducted using clusterProfiler (23) with the threshold p < 0.05 and FDR < 0.25 (BH correction). The results were visualized using bubble plots and network diagrams.

### 2.7 Analysis of protein–protein interactions

Protein–protein interaction (PPI) networks were constructed using the STRING database (28) (minimum interaction score=0.15) and GeneMANIA (29) to identify functionally similar genes. The networks were visualized in Cytoscape (Version 3.10.1) (30).

### 2.8 Regulatory network construction

Transcription factors (TFs) regulating the key genes were retrieved from ChIPBase (31), and miRNA interactions were obtained from TarBase (32). RNA-binding proteins (RBPs) interacting with key genes were predicted using StarBase v3.0 (33). Potential drug targets were identified using the Comparative Toxicogenomics Database (34). All networks were visualized in Cytoscape (30).

### 2.9 Expression, correlation, and diagnostic analysis of key genes

Differences in key gene expression between the HF and control groups were analyzed using the Wilcoxon rank-sum test for both the GSE57338 and GSE76701 datasets. ROC curves were generated to evaluate diagnostic performance. Spearman correlation analysis was performed to assess pairwise correlations between key genes, with results visualized using heatmaps and scatter plots (pheatmap, ggplot2).

### 2.10 Immune infiltration analysis

Single-sample gene set enrichment analysis (ssGSEA) (22) was performed to quantify the relative abundance of 28 immune cell types (35) in GSE57338. Differences in immune infiltration between high-risk and low-risk groups were evaluated. Spearman correlation analysis was used to assess correlations between key genes and immune cells, which were visualized using bubble plots.

### 2.11 Analysis of high- and low-risk groups

Based on the median risk score, the samples from heart failure patients in GSE57338 were divided into high-risk and low-risk groups, which were then subjected to differential expression analysis using limma. GSEA and GSVA were conducted as described in section 2.3 to identify differences in functional pathways.

### 2.12 Statistical analysis

All analyses were performed in R (Version 4.3.0). For continuous variables, Student's t-test and the Mann–Whitney U test were used in the case of normally distributed and non-normally distributed data, respectively. Spearman correlation analysis was applied for assessing correlations. A two-sided p-value < 0.05 was considered statistically significant.

## 3 Results

### 3.1 Data preprocessing

After normalization, inter-sample batch effects were eliminated in both datasets (Supplementary Figure S2A–D). Principal component analysis (PCA) of the normalized expression matrix for GSE57338 revealed a clear separation between HF and control samples along the first principal component, indicating substantial transcriptomic differences between the two groups (Figure 1). GSE57338 contained 177 samples from heart failure patients and 136 control samples, whereas GSE76701 contained 4 samples from heart failure patients and 4 control samples (Supplementary Table S1). The complete list of GMFRGs (1,729 genes) is provided in Supplementary Table S1.

**Figure 1:**
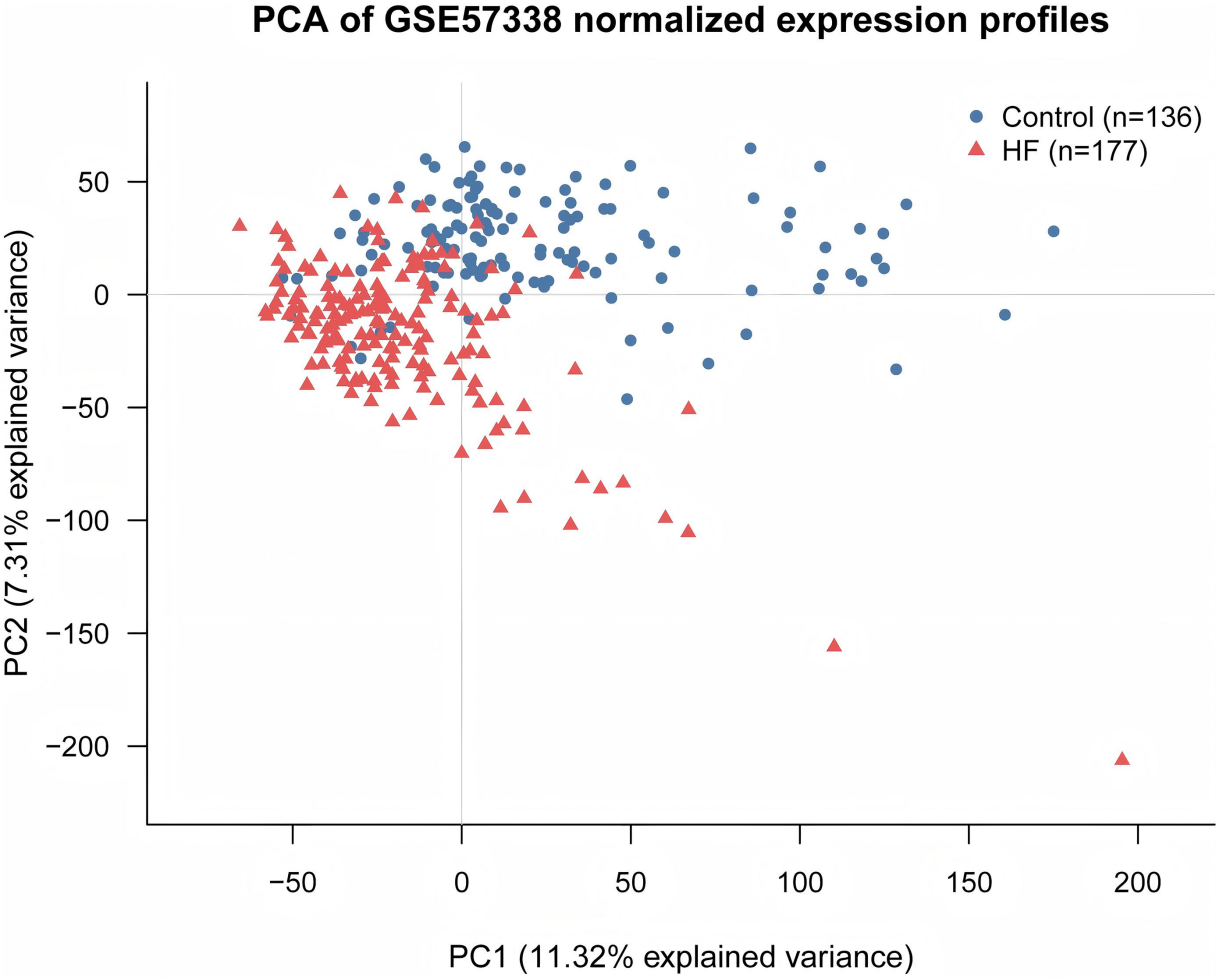
Principal component analysis (PCA) of the GSE57338 dataset based on the normalized expression matrix. Samples are colored by group (red: heart failure, blue: control). The first two principal components (PC1 and PC2) are shown, with the percentage of variance explained indicated in parentheses. The PCA plot demonstrates the overall transcriptional separation between HF and control samples.

Dataset GSE57338 before (A) and after (B) normalization; Dataset GSE76701 before (C) and after (D) normalization.

### 3.2 Identification of gMFRDEGs

Differential expression analysis identified 45 DEGs, 24 upregulated and 21 downregulated (Figure 2A). The intersection of 1,791 GRGs and 3,339 MFRGs yielded 1,729 GMFRGs, among which 14 genes overlapped with DEGs (Figure 2B). These 14 GMFRDEGs are shown in the heatmap in Figure 2C. The complete list of GMFRGs is provided in Supplementary Table S1.

**Figure 2:**
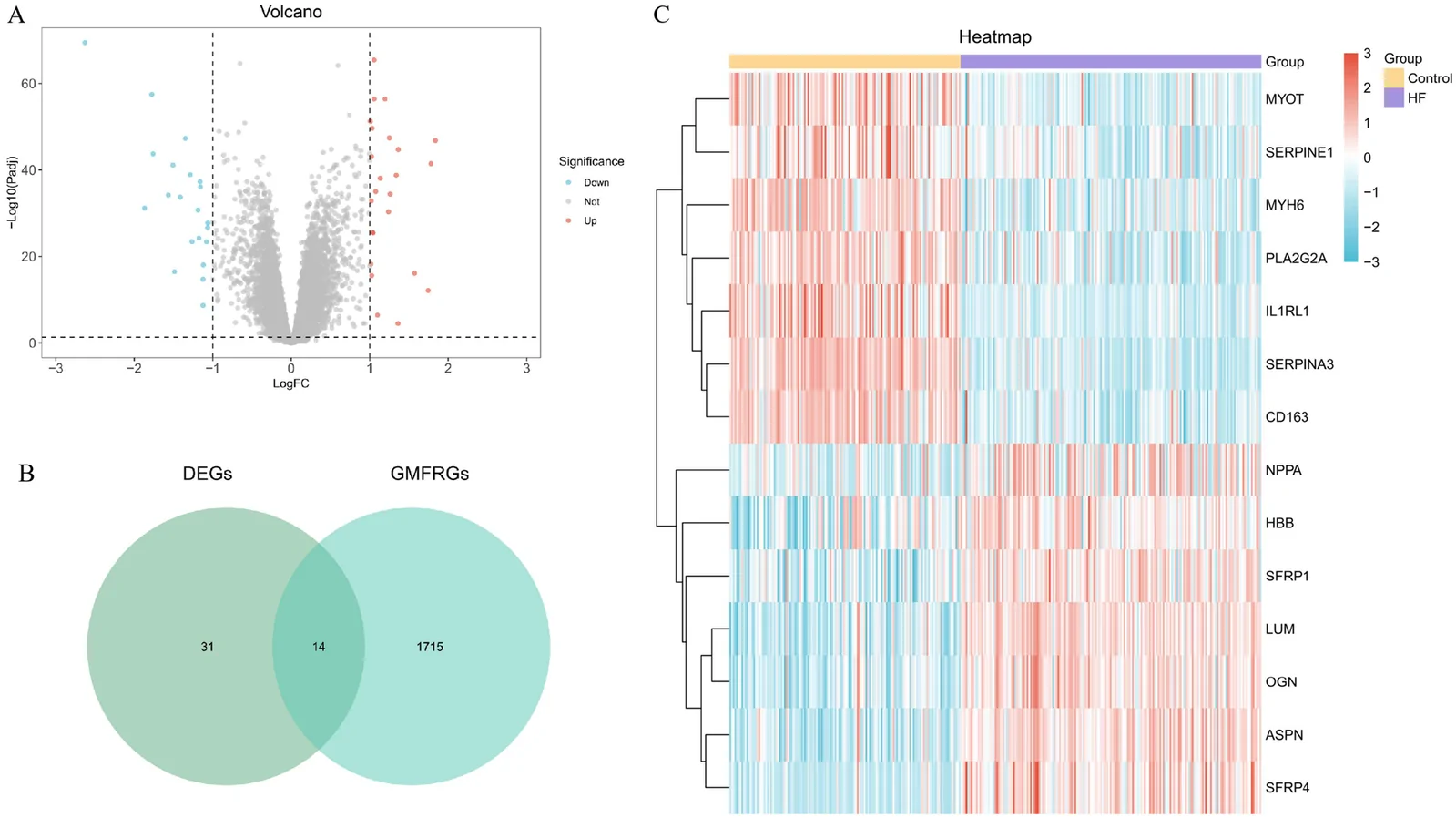
Differential expression analysis. **(A)** Volcano plot of differentially expressed genes (DEGs) between the HF and control groups in dataset GSE57338. **(B)** Venn diagram showing the intersection between DEGs and glycolysis- and myocardial fibrosis-related genes (GMFRGs) in dataset GSE57338. **(C)** Heatmap of glycolysis- and myocardial fibrosis-related differentially expressed genes (GMFRDEGs) in dataset GSE57338.

A. Volcano plot of differentially expressed genes (DEGs) between the HF and control groups for dataset GSE57338. B. Venn diagram visualizing DEGs and glycolysis- and myocardial fibrosis-related genes (GMFRGs) for dataset GSE57338. C. Heatmap of DEGs related to glycolysis and myocardial fibrosis (GMFRDEGs) in dataset GSE57338.

### 3.3 Gene set enrichment and gene set variation analyses

GSEA showed significant enrichment of Marzec Il2 signaling dn and Stambolsky targets of mutated tp53 dn (Figures 3A, B). GSVA identified positive enrichment of Medicus variant lgh mmsef fusion to transcriptional activation and negative enrichment of Nectin necl trans heterodimerization (Figures 3C, D). Similar results were observed for dataset GSE76701 (Figure 3E–J). The complete results are included in Supplementary Tables S2–S5.

**Figure 3:**
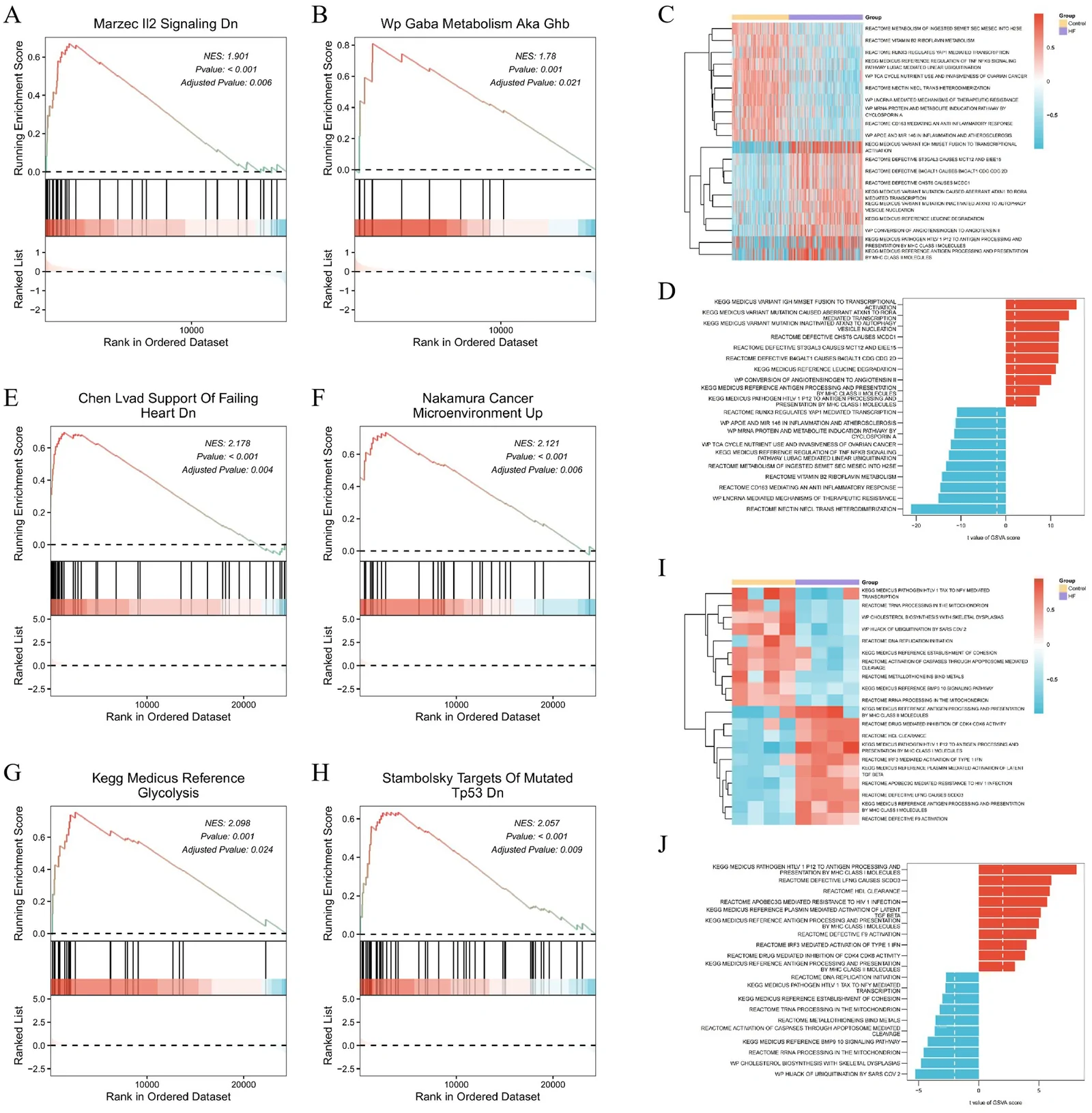
Gene set enrichment analysis (GSEA) and gene set variation analysis (GSVA). **A**. Enrichment plot for Marzec Il2 signaling dn (NES=1.901, p=0.006). **B**. Enrichment plot for Gaba metabolism aka ghb (NES=1.780, p=0.021). **C,D**. Heatmap **(C)** and bidirectional bar plot **(D)** of GSVA results for dataset GSE57338 (HF vs. Control). **E**. Enrichment plot for Chen Lvad support of failing heart dn (NES=2.178, p=0.004). **F**. Enrichment plot for Nakamura cancer microenvironment up (NES=2.121, p=0.006). **G**. Enrichment plot for Medicus reference glycolysis (NES=2.098, p=0.024). **H**. Enrichment plot for Stambolsky targets of mutated tp53 dn (NES=2.057, p=0.009). **I,J**. Heatmap **(I)** and bidirectional bar plot **(J)** of GSVA results for the GSE76701 validation dataset (HF vs. Control). All enrichment plots were generated using the clusterProfiler package, and GSVA was performed using the GSVA package with the c2.cp.v2023.2.Hs.symbols gene set.

### 3.4 Machine learning and identification of key genes

Logistic regression identified 14 GMFRDEGs genes (p < 0.05, Figure 4A). Subsequent analysis via the SVM-RFE algorithm showed the highest accuracy for five genes (Figures 4B, C): *SERPINA3*, *HBB*, *NPPA*, *LUM*, and *OGN* (Figure 4D). LASSO regression confirmed this optimal feature gene set (Figures 4E, F).

**Figure 4:**
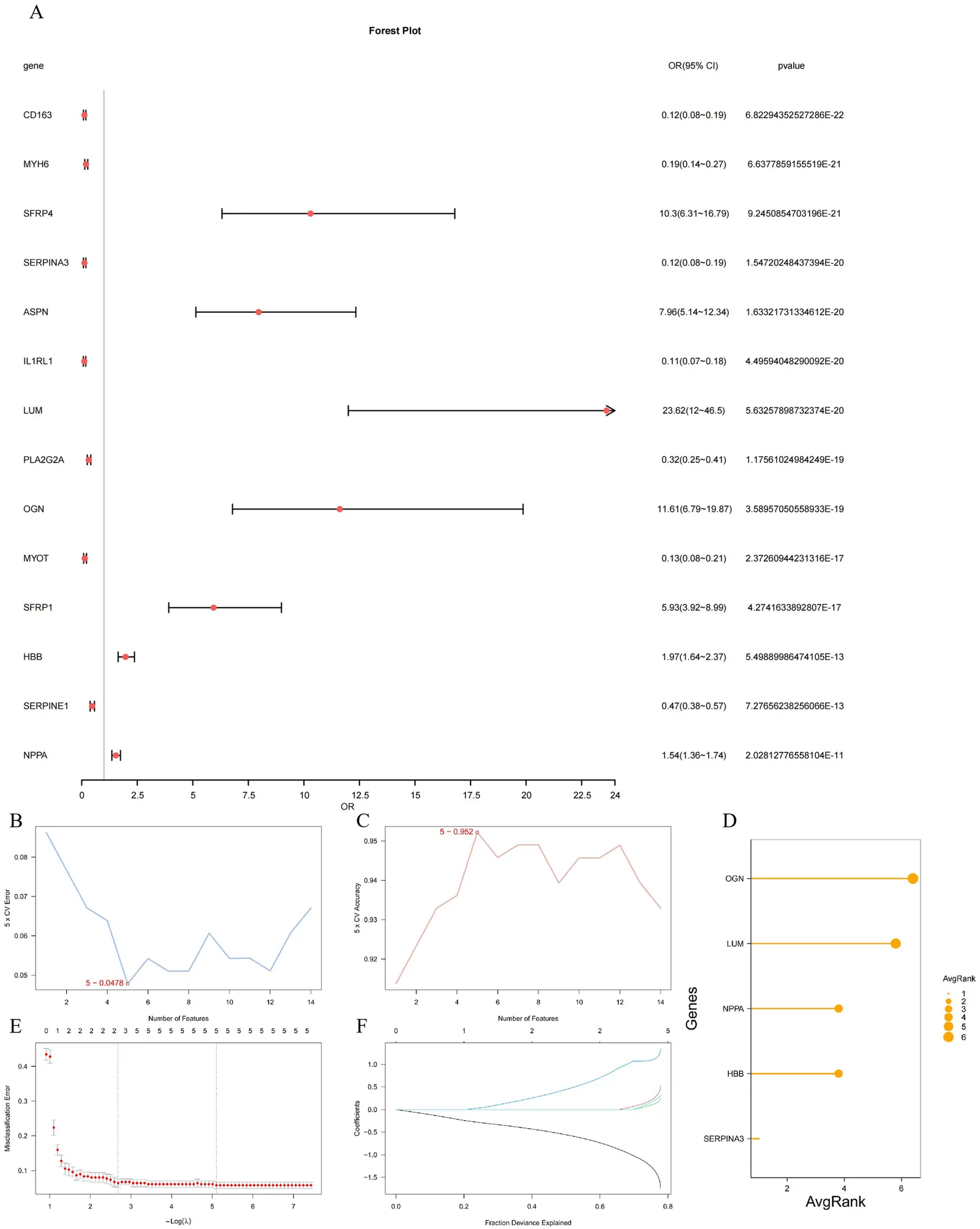
Forest plot and analysis via machine learning. **A**. Forest plot of 14 glycolysis- and myocardial fibrosis-related differentially expressed genes (GMFRDEGs) with p value < 0.05 in the logistic regression model. **B,C**. Visualization of the number of genes with the lowest error rate **(B)** and highest accuracy **(C)** obtained using the SVM-RFE model. **D**. Lollipop plot showing the average importance ranking for the top five genes with the highest accuracy obtained via the SVM-RFE model. **E,F**. Diagnostic model plot **(E)** and variable trajectory plot **(F)** of the LASSO regression model.

### 3.5 Diagnostic model construction and validation

A logistic regression model was constructed based on the five key genes identified. In the training set (GSE57338), the model achieved an excellent diagnostic performance (AUC=0.983, Figure 5A). Among individual genes, SERPINA3 showed the highest AUC (0.998, Figure 6D), exceeding that of the combined model; the latter was nevertheless retained for its integrative coverage of multiple HF-related biological pathways.The calibration curve showed good agreement between predicted and observed probabilities (Figure 5B), and DCA confirmed clinical utility (Figure 5C). The nomogram visualized the contribution of each key gene (Figure 5D). Analysis of functional similarity (Friends) identified *NPPA* as a critical gene in HF (Figure 5E). When applied to the validation set (GSE76701), the model maintained a robust performance (AUC=0.938, Figure 5F), with DCA confirming its clinical applicability (Figure 5G).

**Figure 5:**
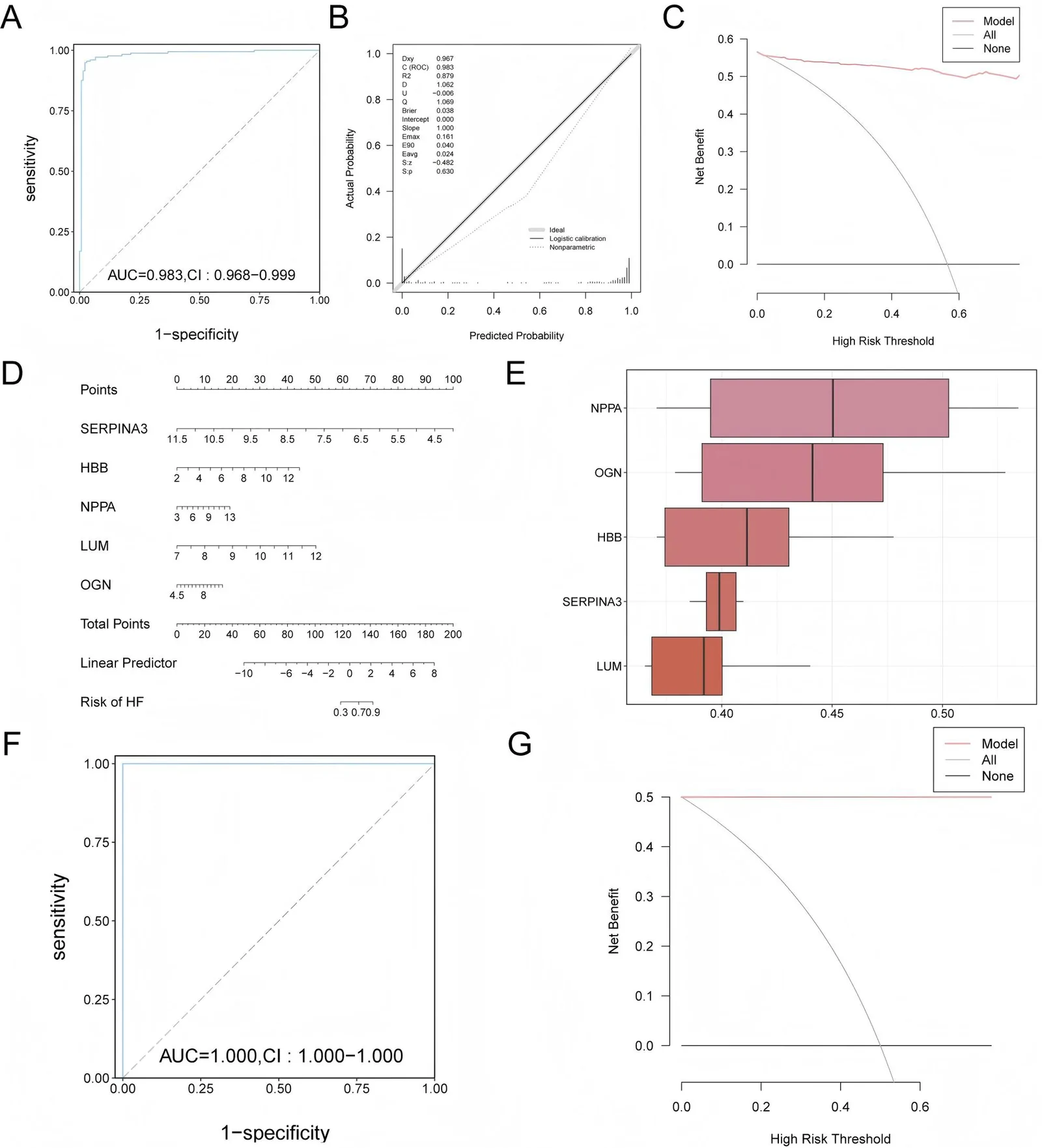
Diagnostic and validation analysis of the HF model. **A**. Receiver operating characteristic (ROC) curve of the logistic regression model for dataset GSE57338 (AUC=0.983, 95% CI: 0.968–0.999). **B,C**. Calibration curve plot **(B)** and decision curve analysis (DCA) **(C)** for the HF diagnostic model. **D**. Nomogram of the key genes. **E**. Functional similarity (Friends) analysis of key genes. **F,G**. ROC curve **(F)** and DCA **(G)** for the external validation dataset GSE76701. AUC > 0.9 indicates high accuracy; DCA *y*-axis: net benefit, *x*-axis: threshold probability. The ROC curves were generated using the pROC package, calibration curves were plotted using the rms package, and DCA was performed using the ggDCA package.

**Figure 6:**
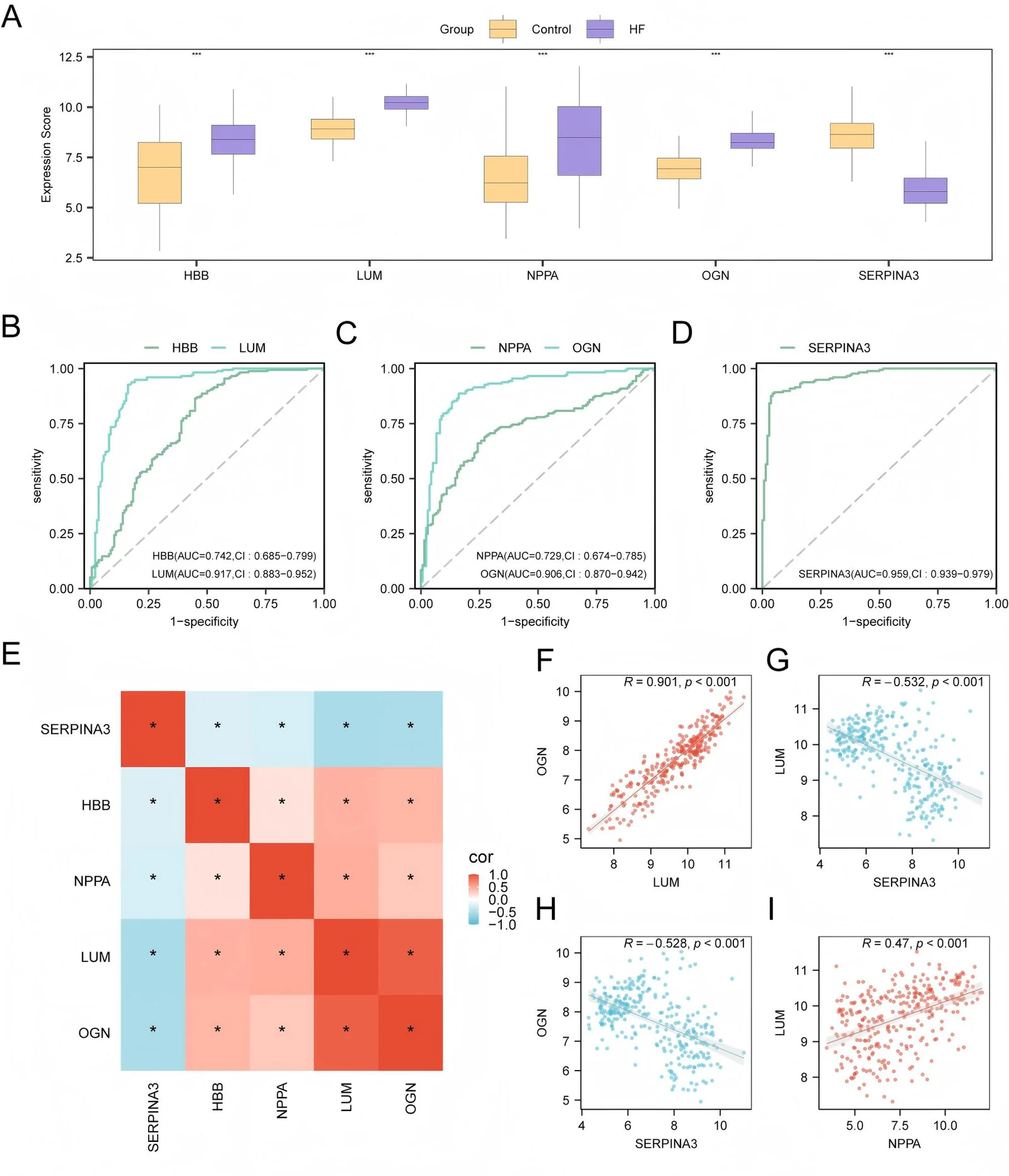
Differential expression, correlation analysis, and receiver operating characteristic (ROC) curves for key genes in the GSE57338 dataset. **A**. Comparison of key genes between the HF and control groups in dataset GSE57338 (purple: HF, yellow: control). **B–D**. ROC curves for *HBB* (AUC=0.831, 95% CI: 0.781–0.881) and *LUM* (AUC=0.982, 95% CI: 0.968–0.997) **(B)**, *NPPA* (AUC=0.866, 95% CI: 0.822–0.910) and *OGN* (AUC=0.943, 95% CI: 0.916–0.970) **(C)**, and *SERPINA3* (AUC=0.998, 95% CI: 0.995–1.000) **(D)** in dataset GSE57338. **E**. Correlation heatmap of key genes (with red and blue indicating positive and negative correlations, respectively, and color intensity denoting the correlation strength). **F–I**. Scatter plots of pairwise correlations between *LUM* and *OGN* **(F)**, *LUM* and *SERPINA3* **(G)**, *OGN* and *SERPINA3* **(H)**, and *NPPA* and *LUM* **(I)**. Significance: ns *p* ≥ 0.05; * p < 0.05; ** p < 0.01; *** p < 0.001. AUC > 0.9: high accuracy. |r|: < 0.3 (weak/no correlation), 0.3–0.5 (weak), 0.5–0.8 (moderate), > 0.8 (strong).

### 3.6 Functional enrichment of key genes

GO and KEGG enrichment analyses showed that the five key genes were primarily involved in blood pressure regulation, vascular processes, and extracellular matrix organization (Figure 7A–E). The complete results are included in Supplementary Table S6.

**Figure 7:**
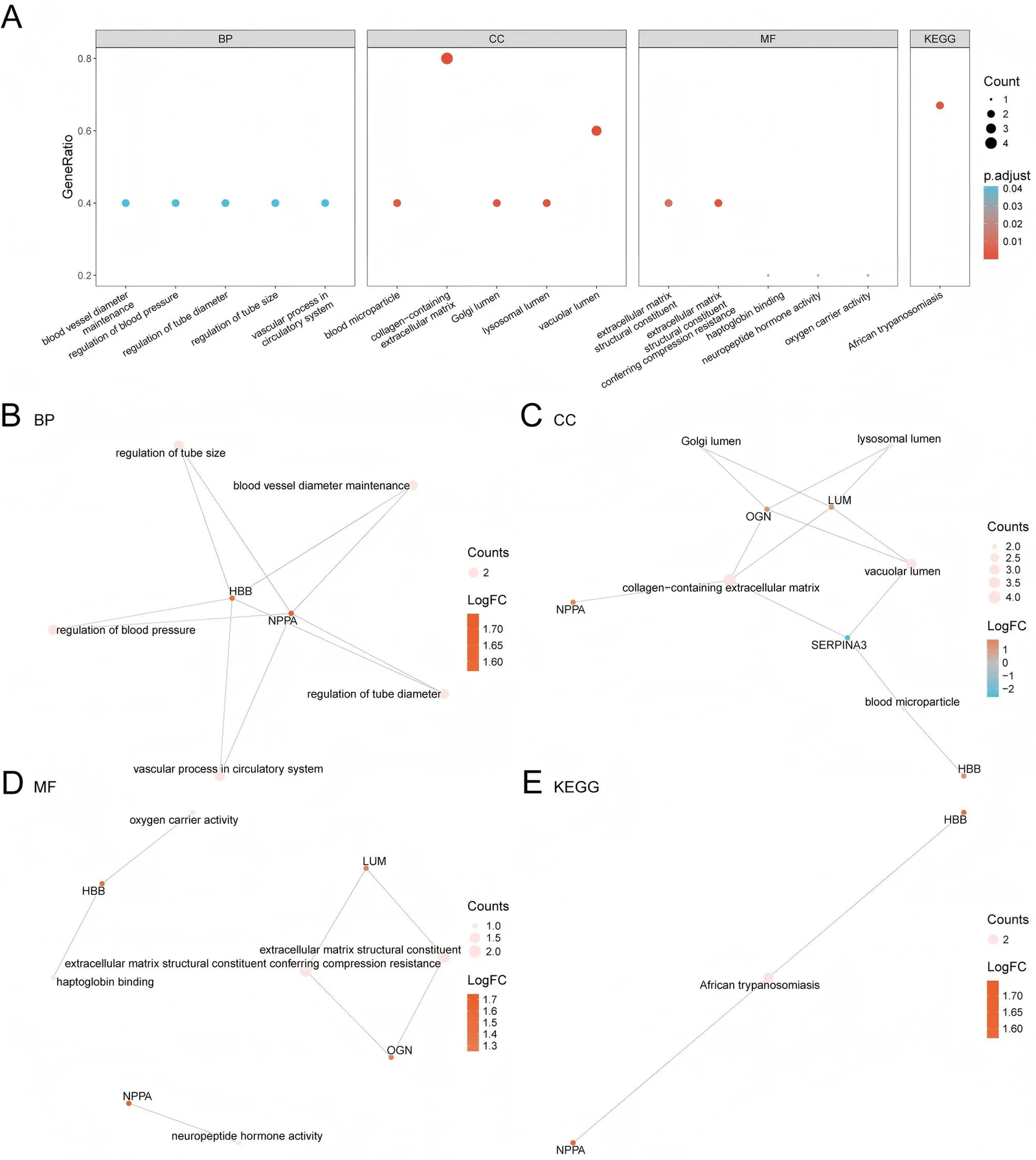
Go and kEGG enrichment analysis of the key genes. **A**. Bubble plot of GO terms, i.e., biological processes (BP), cellular components (CC), and molecular functions (MF), and KEGG pathways. The bubble size and color indicate the gene count and adjusted p-value (red: lower p adjust), respectively. **(B–E)** Network plots for BP **(B)**, CC **(C)**, MF **(D)**, and KEGG **(E)** The pink nodes denote GO terms, whereas the lines indicate term–gene relationships. The red and blue nodes indicate upregulated and downregulated genes, respectively. Significance threshold: p adjust < 0.05 and FDR < 0.25 (BH correction).

### 3.7 Protein–protein interaction and regulatory networks

A PPI network for the five key genes was constructed using the STRING database and GeneMANIA, revealing interactions between them and 20 functionally similar proteins (Figures 8A,B). Multi-level regulatory networks were also constructed. The mRNA–TF network contained 4 mRNAs and 19 TFs (Figure 8C). The mRNA–miRNA network comprised 2 mRNAs and 35 miRNAs (Figure 8D). The mRNA–RBP network included 1 mRNA (*SERPINA3*) and 23 RBPs (Figure 8E). A total of 4 mRNAs and 26 potential drugs or compounds were identified in the mRNA–drug network (Figure 8F). The complete networks are included in Supplementary Tables S7–S10.

**Figure 8:**
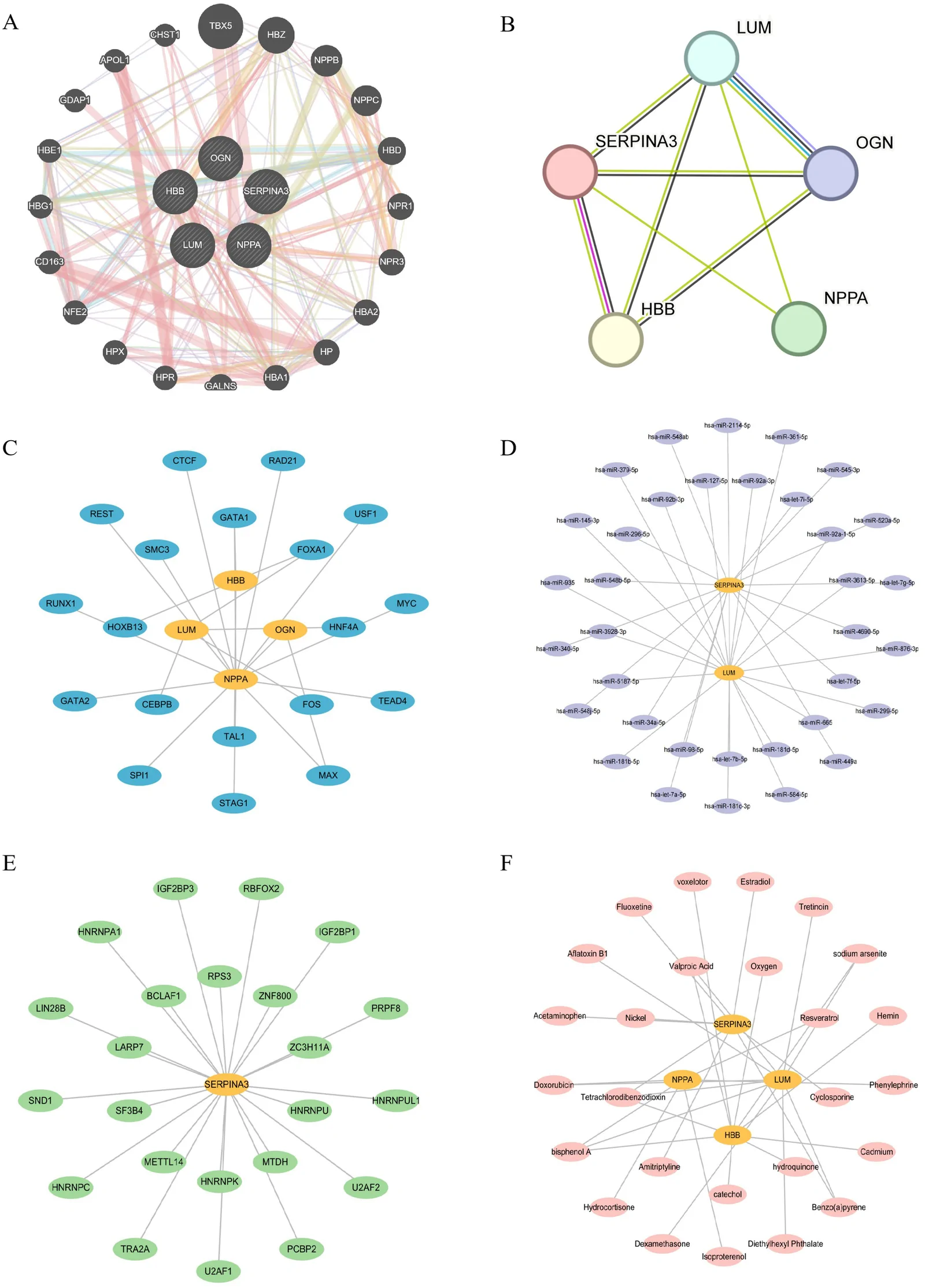
Analysis of protein–protein interactions and regulatory networks for key genes. **A**. Interaction network of functionally similar genes predicted using GeneMANIA. The circles indicate key genes and functionally similar genes, whereas colored lines denote interrelated functions. **B**. Protein–protein interaction (PPI) network for key genes based on the STRING database. **C**. mRNA–TF regulatory network. **D**. mRNA–miRNA regulatory network. **E**. mRNA–RBP regulatory network. **F.** mRNA–drug regulatory network for key genes. Color code: orange, mRNA; blue, transcription factor (TF); purple/gray, miRNA; green, RNA-binding protein (RBP); and pink, drug.

### 3.8 Expression, diagnostic performance, and correlation analysis of key genes (Gse57338)

All five key genes were significantly upregulated in the HF group (p < 0.001, Figure 6A). *LUM, OGN,* and *SERPINA3* showed high diagnostic accuracy (AUC > 0.9), whereas *HBB* and *NPPA* showed moderate diagnostic accuracy (Figure 6B–D). Strong positive correlations were observed among key genes, especially between *LUM* and *OGN* (r=0.901, Figure 6E–I).

### 3.9 Expression, diagnostic performance, and correlation analysis of key genes (Gse76701)

*HBB* and *SERPINA3* were significantly upregulated (p < 0.05, Figure 9A) and maintained a high diagnostic accuracy (AUC > 0.9). *LUM* and *NPPA* showed a moderate diagnostic accuracy, whereas *OGN* showed a lower diagnostic accuracy (Figure 9B–D). The correlation between *LUM* and *OGN* remained strong (r=0.857, Figure 9E–I).

**Figure 9:**
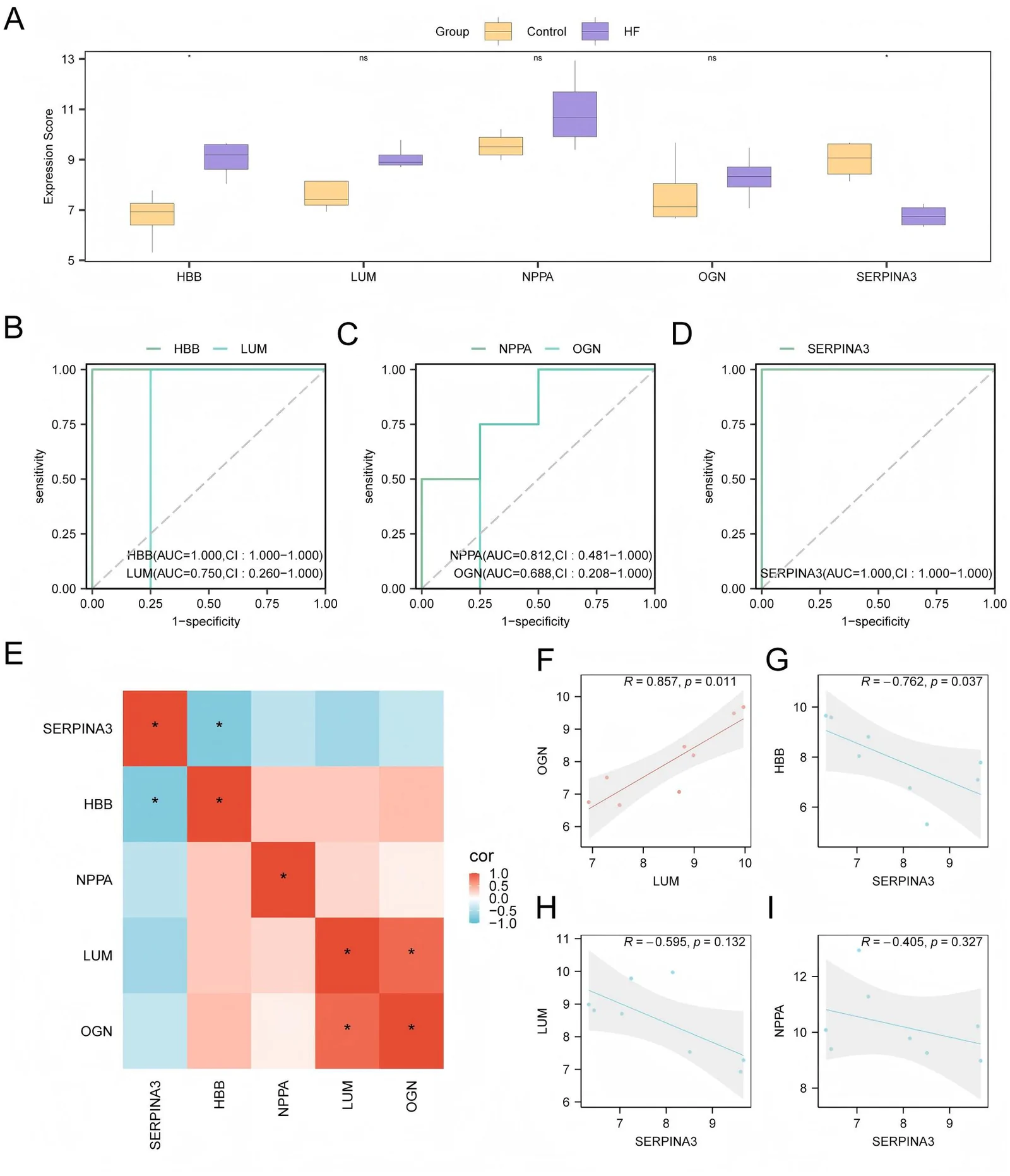
Differential expression, correlation analysis and receiver operating characteristic (ROC) curves for key genes in the GSE76701 dataset. **A**. Comparison of key genes between the HF and control groups (purple: HF, yellow: control). **B–D**. ROC curves for *HBB* (AUC=1.000, 95% CI: 1.000–1.000) and *LUM* (AUC=0.875, 95% CI: 0.515–1.000) **(B)**, *NPPA* (AUC=0.813, 95% CI: 0.438–1.000) and *OGN* (AUC=0.688, 95% CI: 0.208–1.000) **(C)**, and *SERPINA3* (AUC=1.000, 95% CI: 1.000–1.000) **(D) E** Correlation heatmap of key genes (with red and blue indicating positive and negative correlations, respectively, and color intensity denoting the correlation strength). **F–I**. Scatter plots of correlations between *OGN* and *LUM* **(F)**, *HBB* and *SERPINA3* **(G)**, *LUM* and *SERPINA3* **(H)**, and *NPPA* and *SERPINA3* **(I)**. Significance: ns *p* ≥ 0.05; * p < 0.05; ** p < 0.01; *** p < 0.001. AUC > 0.9: high accuracy. |r|: < 0.3 (weak/no correlation), 0.3–0.5 (weak), 0.5–0.8 (moderate), > 0.8 (strong).

### 3.10 Immune infiltration analysis

A total of 11 immune cell types with significantly different infiltration levels were identified by ssGSEA between the high-risk and low-risk groups (p < 0.05, Figure 10A). In the LowRisk group, activated dendritic cells and myeloid-derived suppressor cells showed the strongest positive correlation (r=0.863, p < 0.05, Figure 10B), and *SERPINA3* correlated most strongly with central memory CD8 T cells (r=0.748, p < 0.05, Figure 10D). In the high-risk group, central memory CD8 T cells and T follicular helper cells showed the strongest positive correlation (r=0.692, p < 0.05, Figure 10C), and *LUM* correlated most strongly with regulatory T cells (r=0.672, p < 0.05, Figure 10E).

**Figure 10:**
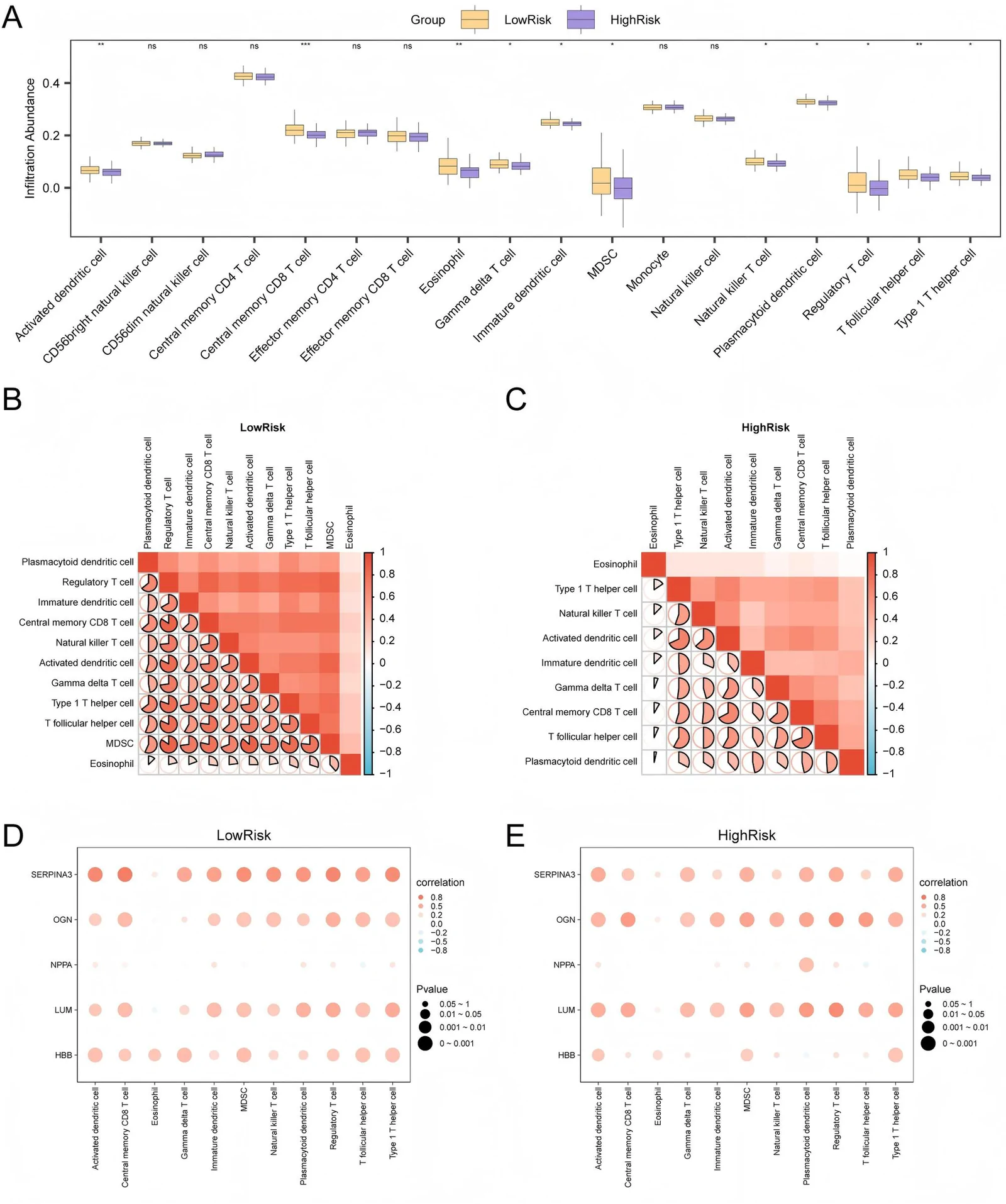
Immune infiltration analysis by single-sample gene set enrichment analysis (ssGSEA). **A**. Comparison of immune cells between the low-risk and high-risk groups within the HF group (purple: high-risk, yellow: low-risk). **B,C**. Correlation matrices for the abundance of infiltrating immune cells in the low-risk **(B)** and high-risk **(C)** groups (with red and blue indicating positive and negative correlations, respectively, and color intensity denoting the correlation strength). **D,E**. Bubble plots of correlations between immune cell abundance and key genes in the low-risk **(D)** and high-risk **(E)** groups. The bubble size indicates p-value intervals (the larger the bubble, the more significant the correlation). Correlation strength (|r|): <0.3 (weak/no correlation), 0.3–0.5 (weak), 0.5–0.8 (moderate), > 0.8 (strong). Significance: ns *p* ≥ 0.05; * p < 0.05; ** p < 0.01; *** p < 0.001.

### 3.11 Gsea and gsva for the high-risk and low-risk groups

GSEA identified differences in functional pathways between the high-risk and low-risk groups. The overall distribution of enriched pathways is shown in the mountain plot in Figure 11A. The results of GSEA revealed that genes in the high-risk group were significantly enriched in Medicus reference Wnt signaling modulation Sost Lrp4 (Figure 11B), Sakai tumor infiltrating monocytes up (Figure 11C), Dilated cardiomyopathy (Figure 11D), and Yauck Hedgehog signaling paracrine dn (Figure 11E). GSVA further identified differentially enriched pathways (Figure 12A,B). The complete results are included in Supplementary Tables S11, S12.

**Figure 11:**
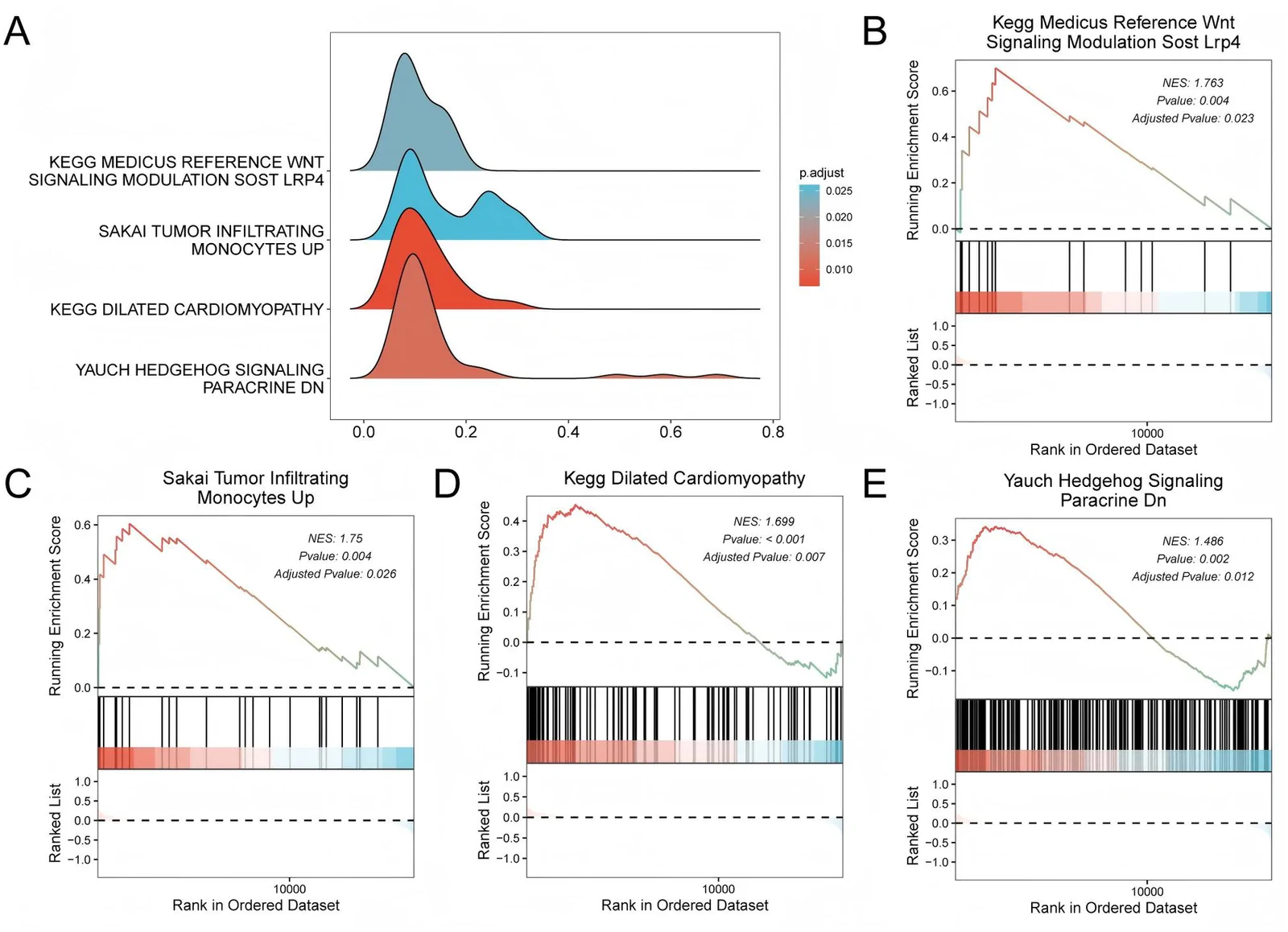
Gene set enrichment analysis for dataset GSE57338 **A**. Mountain plot of enriched biological functions in the HF group for dataset GSE57338 (color: adjusted p-value, red: smaller, blue: larger). **B–E**. Enrichment plots showing significant enrichment of Medicus reference Wnt signaling modulation Sost Lrp4 **(B)**, Sakai tumor infiltrating monocytes up **(C)**, Dilated cardiomyopathy **(D)**, and Yauck Hedgehog signaling paracrine dn **(E)** Significance threshold: adjusted p < 0.05 and FDR < 0.05 (BH correction).

**Figure 12:**
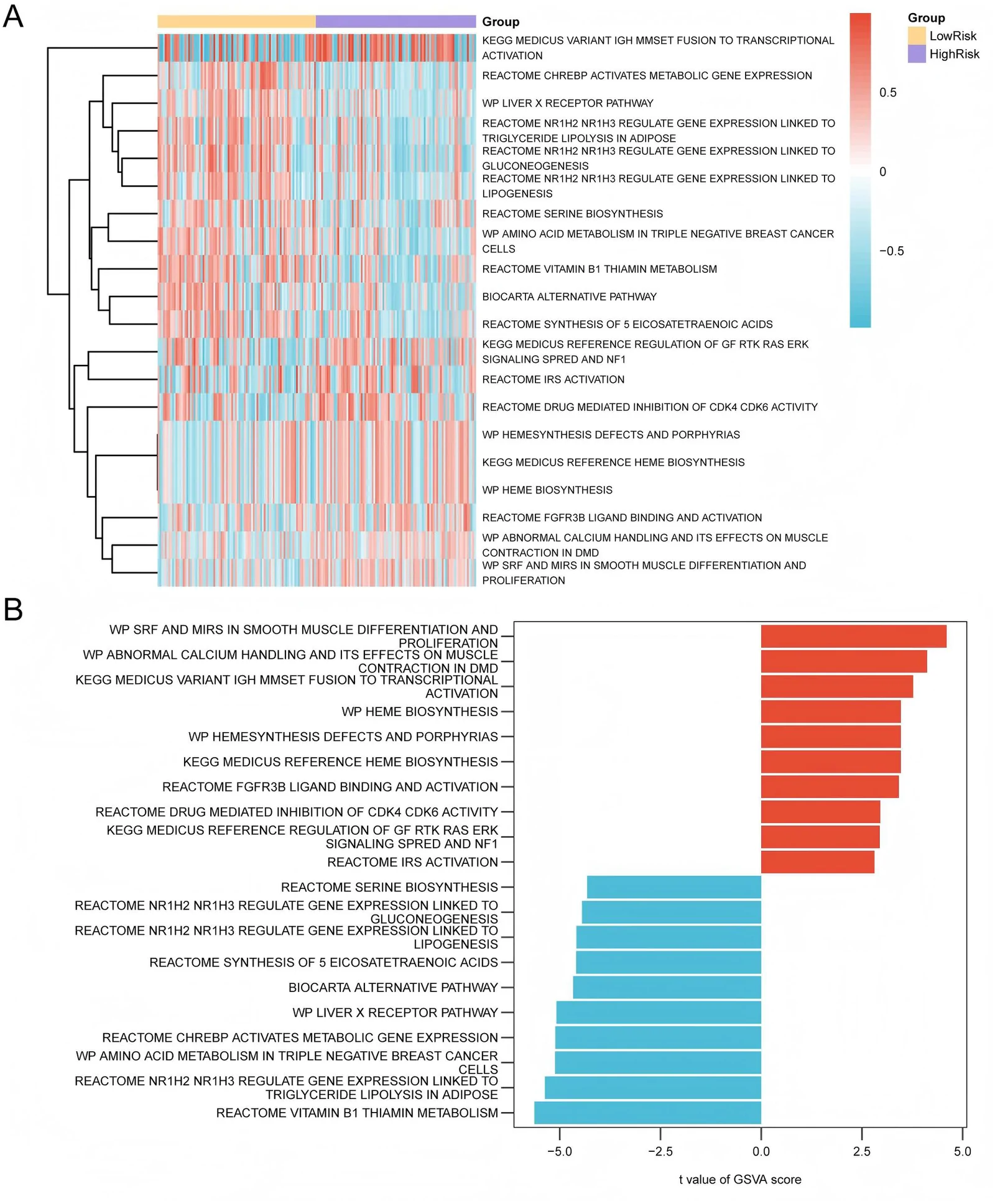
Gene set variation analysis (GSVA) of the high-risk and low-risk groups for dataset GSE57338. **A**. Heatmap of significantly enriched pathways (the blue and red colors indicate low and high enrichment, respectively). **B**. Bidirectional bar plot of GSVA results for the high-risk (purple) and low-risk (yellow) groups. The top 10 positively and top 10 negatively enriched pathways are shown (p < 0.05).

## 4 Discussion

HF affects over 60 million people worldwide, with a 5-year mortality rate exceeding that of many common malignancies. Current diagnostic approaches relying on imaging and natriuretic peptides remain insufficient for early detection and personalized intervention, largely due to the high heterogeneity of HF and the complex interplay between its core drivers, i.e., myocardial energy metabolism reprogramming and myocardial fibrosis. In this study, we identified five key genes (*SERPINA3, HBB, NPPA, LUM*, and *OGN*) associated with glycolysis and myocardial fibrosis in HF and constructed a robust diagnostic model with excellent predictive performance.

Among these, *SERPINA3* demonstrated the highest diagnostic utility (AUC=0.998 in the training set). This gene encodes alpha-1-antichymotrypsin, a serine protease inhibitor recently linked to cardiovascular prognosis. Boen et al. reported that elevated plasma SERPINA3 protein levels were associated with increased all-cause mortality in heart failure patients (36). Mechanistically, *SERPINA3* has been shown to activate NF-KB in myocardial tissue, promoting inflammation, oxidative stress, and apoptosis (37). Our finding that *SERPINA3* was significantly upregulated in the HF group and positively correlated with central memory CD8 T cells in the low-risk subgroup supports these observations by suggesting that this gene may contribute to HF progression not only through direct pro-inflammatory effects but also via modulation of specific T cell subsets. This immune-regulatory role of *SERPINA3* has not been previously described in HF and warrants further mechanistic investigation.

Beyond its role as a diagnostic biomarker, *NPPA* encodes atrial natriuretic peptide (ANP), one of the most extensively studied peptides in cardiovascular physiology. ANP is synthesized primarily in atrial myocytes in response to wall stretch and plays a critical homeostatic role in volume load regulation through natriuresis, diuresis, and vasodilation, while also antagonizing the renin–angiotensin–aldosterone system (RAAS) (37). In HF, elevated ANP levels reflect compensatory activation of this counter-regulatory system, but chronic elevation may also indicate persistent hemodynamic stress and disease severity. Our finding that NPPA was significantly upregulated in HF patients (AUC=0.866 in the training set) reinforces its well-established role as a sensitive indicator of cardiac volume and pressure overload.

*HBB* encodes the *β*-globin subunit of hemoglobin, a protein central to oxygen transport and delivery. In the context of HF, tissue hypoxia is a common consequence of reduced cardiac output and impaired microvascular perfusion, which in turn drives metabolic reprogramming toward glycolysis (the “Warburg effect” in the heart) and promotes fibrotic remodeling through hypoxia-inducible factor (HIF)-mediated pathways. Interestingly, *HBB* expression in cardiac tissue may reflect both adaptive responses to hypoxia (enhanced oxygen delivery) and maladaptive oxidative stress (release of free heme and iron-catalyzed reactive oxygen species). Our observation that *HBB* maintained high diagnostic accuracy (AUC=0.831 in GSE57338 and AUC=1.000 in GSE76701) suggests that this gene may serve as a sensitive indicator of the hypoxic–glycolytic shift in failing myocardium, although the extreme AUC in the small validation cohort warrants cautious interpretation.

*LUM* and *OGN* emerged as two strongly correlated genes (r=0.901 in GSE57338, r=0.857 in GSE76701) with high diagnostic accuracy. Both gene encode small leucine-rich proteoglycans involved in extracellular matrix organization, a critical process in cardiac fibrosis. Our finding that *LUM* was positively correlated with regulatory T cells in the high-risk group (r=0.672) aligns with recent evidence positioning this gene at the intersection of apoptosis, inflammation, and cellular communication in HF (38) and further suggests that it may promote a pro-fibrotic immune microenvironment by recruiting or expanding regulatory T cells. With regard to *OGN*, Zuo et al. demonstrated that *OGN* deficiency exacerbates angiotensin II-induced cardiac fibrosis, whereas *OGN* overexpression attenuates myofibroblast proliferation by antagonizing LPA3/MMP2/EGFR signaling (39). Consistently, our study identified *OGN* as a key protective gene in HF, suggesting that therapeutic strategies aimed at enhancing its expression or activity could be particularly beneficial for heart failure patients with a prominent fibrotic phenotype.

Importantly, the five-gene combination model demonstrated excellent diagnostic performance (AUC=0.983 in the training set). Notably, *SERPINA3* alone achieved the highest individual diagnostic accuracy (AUC=0.998), which was numerically higher than that of the combined model. This finding highlights SERPINA3 as a particularly promising single biomarker. However, the combined model offers a complementary advantage by integrating multidimensional pathophysiological information—inflammation (*SERPINA3*), hypoxic stress (*HBB*), neurohormonal activation (*NPPA*), and extracellular matrix remodeling (*LUM*/*OGN*)—thereby providing a more comprehensive molecular portrait of HF than any single gene alone. The slightly lower AUC of the combined model is offset by its broader biological coverage and potentially greater stability across heterogeneous patient populations. This improvement reflects the complementary biological information captured by each gene: *SERPINA3* reflects inflammation and immune modulation, *HBB* captures hypoxic/metabolic stress, *NPPA* indicates neurohormonal activation and volume overload, and *LUM/OGN* jointly represent extracellular matrix remodeling and fibrotic processes. The combination of these multidimensional pathway readouts into a single risk score thus provides a more comprehensive molecular portrait of HF pathophysiology than any individual biomarker alone, supporting the clinical utility of multi-marker diagnostic panels.

GO and KEGG enrichment analyses revealed that the five key genes were primarily involved in blood pressure regulation, vascular processes, and structural organization of the extracellular matrix. GSEA further identified significant enrichment of glycolytic pathways and dilated cardiomyopathy pathways, supporting the established paradigm that metabolic reprogramming from fatty acid oxidation toward glycolysis is a core driver of HF progression (4, 5). Notably, the intersection of glycolysis- and myocardial fibrosis-related pathways in our analysis is in line with recent experimental evidence showing that Isthmin-1 improves cardiac function by enhancing glycolysis and activating SIRT1 (40). Our results extend this concept by identifying specific molecular nodes (*SERPINA3, LUM, and OGN*) that may link glycolytic dysregulation to fibrotic remodeling, suggesting that the glycolysis–SIRT1 axis could be therapeutically targeted in HF.

Our ssGSEA analysis revealed 11 immune cell types with significantly different infiltration levels between the high-risk and low-risk groups. The positive correlation between *SERPINA3* and central memory CD8 T cells in the LowRisk group suggests that this gene may facilitate the maintenance of protective immune surveillance, potentially contributing to a less severe disease phenotype. In contrast, the strong positive correlation between *LUM* and regulatory T cells in the high-risk group indicates that this gene may promote an immunosuppressive, pro-fibrotic microenvironment, as regulatory T cells are known to secrete TGF-*β* and promote extracellular matrix deposition. This dichotomy supports the existence of a metabolic–immune–fibrosis axis in HF, wherein distinct molecular drivers shape the immune landscape toward either protective or pathogenic outcomes.

To identify upstream regulators and potential therapeutic compounds, we constructed comprehensive regulatory networks, which revealed extensive transcriptional (4 mRNAs, 19 TFs), post-transcriptional (2 mRNAs, 35 miRNAs; 1 mRNA, 23 RBPs), and pharmacological (4 mRNAs, 26 drugs) interactions. Notably, the mRNA–drug network identified voxelotor (an FDA-approved drug for sickle cell disease) as targeting *HBB*, suggesting a potential repurposing opportunity for HF associated with tissue hypoxia. Voxelotor works by increasing hemoglobin's affinity for oxygen, thereby reducing sickling in erythrocytes; in the context of HF, this mechanism could theoretically improve tissue oxygen delivery and alleviate hypoxia-driven glycolytic reprogramming and fibrotic signaling. However, the cardiac effects of voxelotor have not been directly studied, and this hypothesis requires experimental validation. The interaction between doxorubicin and *NPPA* is consistent with the established role of natriuretic peptides in monitoring anthracycline-induced cardiotoxicity (37), as anthracycline chemotherapy is known to elevate NPPA expression as an early marker of cardiac injury, supporting the clinical relevance of our network predictions. Other identified compounds, including those targeting *SERPINA3*, *LUM*, and *OGN*, represent additional candidate agents for future screening, though their effects on HF pathophysiology remain entirely unexplored and require systematic preclinical investigation. These findings provide a valuable resource for prioritizing future experimental validation and studies on drug repurposing in HF, but should be interpreted as hypothesis-generating rather than clinically actionable at this stage.

## 5 Limitations

Our analyses were based solely on publicly available datasets, and independent validation in larger, prospectively collected cohorts is thus necessary. The external validation dataset GSE76701 has a very limited sample size (n=8, with 4 HF and 4 control samples), which severely restricts statistical power and leads to wide confidence intervals for the individual gene AUCs (e.g., OGN: 0.208–1.000; LUM: 0.260–1.000). This small-sample context likely explains the lower diagnostic accuracy of OGN (AUC=0.688) in this cohort, and the extreme AUC values of 1.000 for HBB and SERPINA3 should also be interpreted with caution. Therefore, we emphasize that prospective validation in larger, well-powered cohorts is urgently needed before any clinical implementation can be considered. Our study is purely computational; therefore, experimental validation through animal models or *in vitro* functional assays is required to establish causal relationships between the identified key genes and HF pathogenesis. Fourth, while logistic regression was selected for its clinical interpretability and direct estimation of odds ratios, we acknowledge that we did not perform direct comparisons with other machine-learning algorithms such as Random Forest (RF) or XGBoost. Given that our primary goal was to develop a clinically applicable and interpretable diagnostic score, logistic regression remains the most straightforward and widely accepted choice in clinical biomarker studies. Moreover, the current sample size (n=313 in the training set), while adequate for logistic regression with five predictors, may be suboptimal for more complex algorithms that require larger samples to avoid overfitting. Nevertheless, we recognize that comparative analyses with RF or XGBoost in future studies with larger cohorts could further strengthen the justification for model selection. Finally, while we predicted potential drugs targeting these key genes, preclinical and clinical studies are still required to determine their safety and efficacy for HF treatment. Future studies integrating multi-omics analyses and large-scale prospective cohorts will help address these limitations and facilitate clinical translation.

## 6 Conclusions

The five key genes (*SERPINA3*, *HBB*, *NPPA*, *LUM*, and *OGN*) identified in this study not only serve as promising diagnostic biomarkers for HF but also highlight the potential therapeutic value of targeting the glycolysis–fibrosis crosstalk. The observed association between these genes and immune infiltration suggests that modulating the metabolic–immune–fibrosis axis may represent a novel therapeutic strategy. Future studies should prioritize experimental validation of these genes in animal models and clinical cohorts to facilitate their translation into clinical practice.

## Data Availability

The datasets presented in this study can be found in online repositories. The names of the repository/repositories and accession number(s) can be found in the article/Supplementary Material.
