# Supplementary material for: Identification of glycolysis- and myocardial fibrosis-related diagnostic biomarkers in heart failure: an integrative bioinformatics and machine learning study
Source: Front Cardiovasc Med. 2026 Sep 22;13:1912375. doi: 10.3389/fcvm.2026.1912375 (PMC13638659; doi:10.3389/fcvm.2026.1912375)
Supplement: Supplementary file 1 [file Image1.pdf]

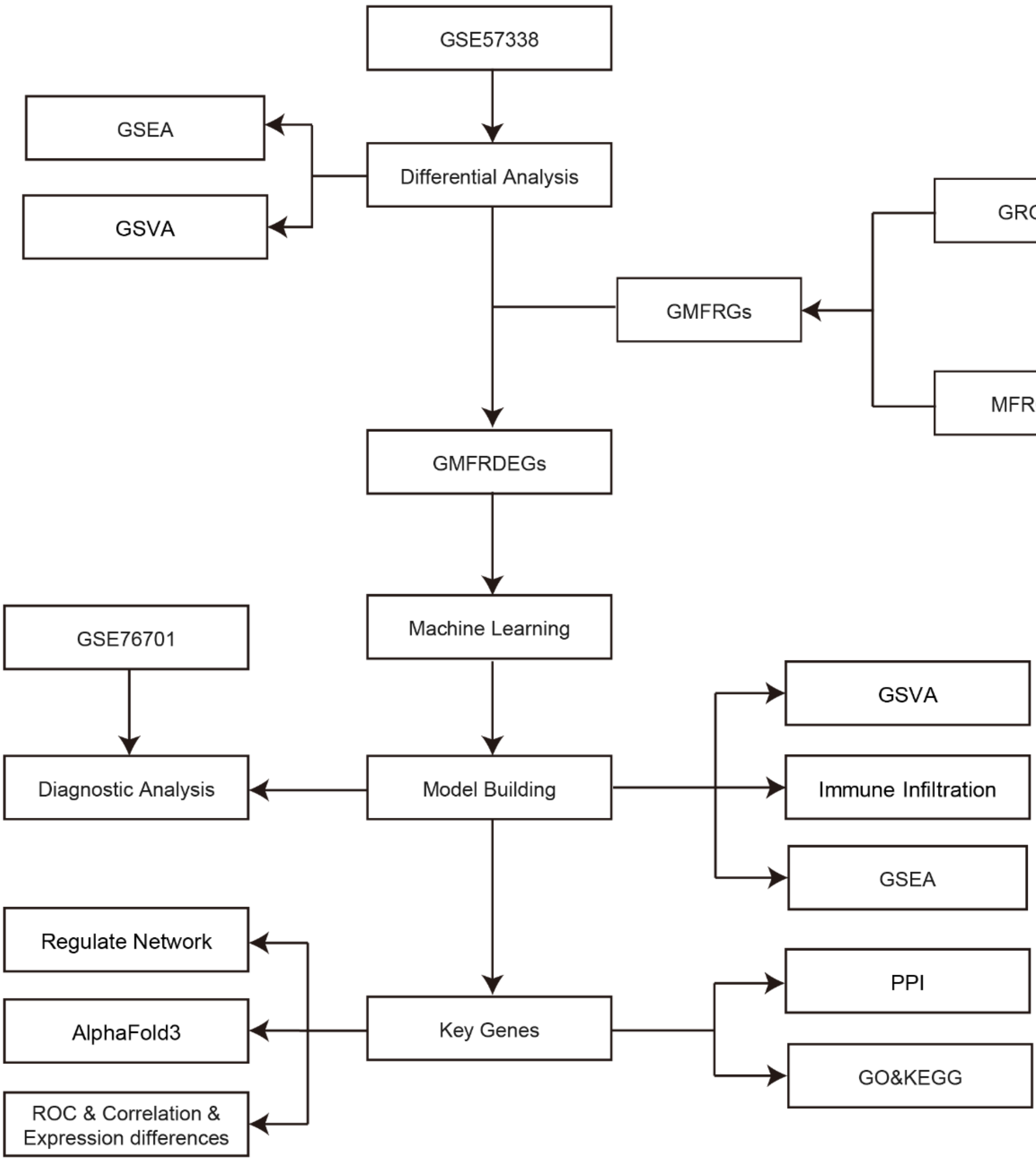

Supplementary Fig.1 Technical route of the study

GO, Gene Ontology; KEGG, Kyoto Encyclopedia of Genes and Genomes; GRGs, Glycolysis Related Genes; MFRGs, Myocardial Fibrosis-Related Genes; GMFRDEGs, Glycolysis and Myocardial Fibrosis-Related Differentially Expressed Genes; GSEA, Gene Set Enrichment Analysis; GSVA, Gene Set Variation Analysis; ROC, Receiver Operating Characteristic; PPI, Protein-protein Interaction.
