# Supplementary material for: Identification of glycolysis- and myocardial fibrosis-related diagnostic biomarkers in heart failure: an integrative bioinformatics and machine learning study
Source: Front Cardiovasc Med. 2026 Sep 22;13:1912375. doi: 10.3389/fcvm.2026.1912375 (PMC13638659; doi:10.3389/fcvm.2026.1912375)
Supplement: Supplementary file 2 [file Image2.pdf]

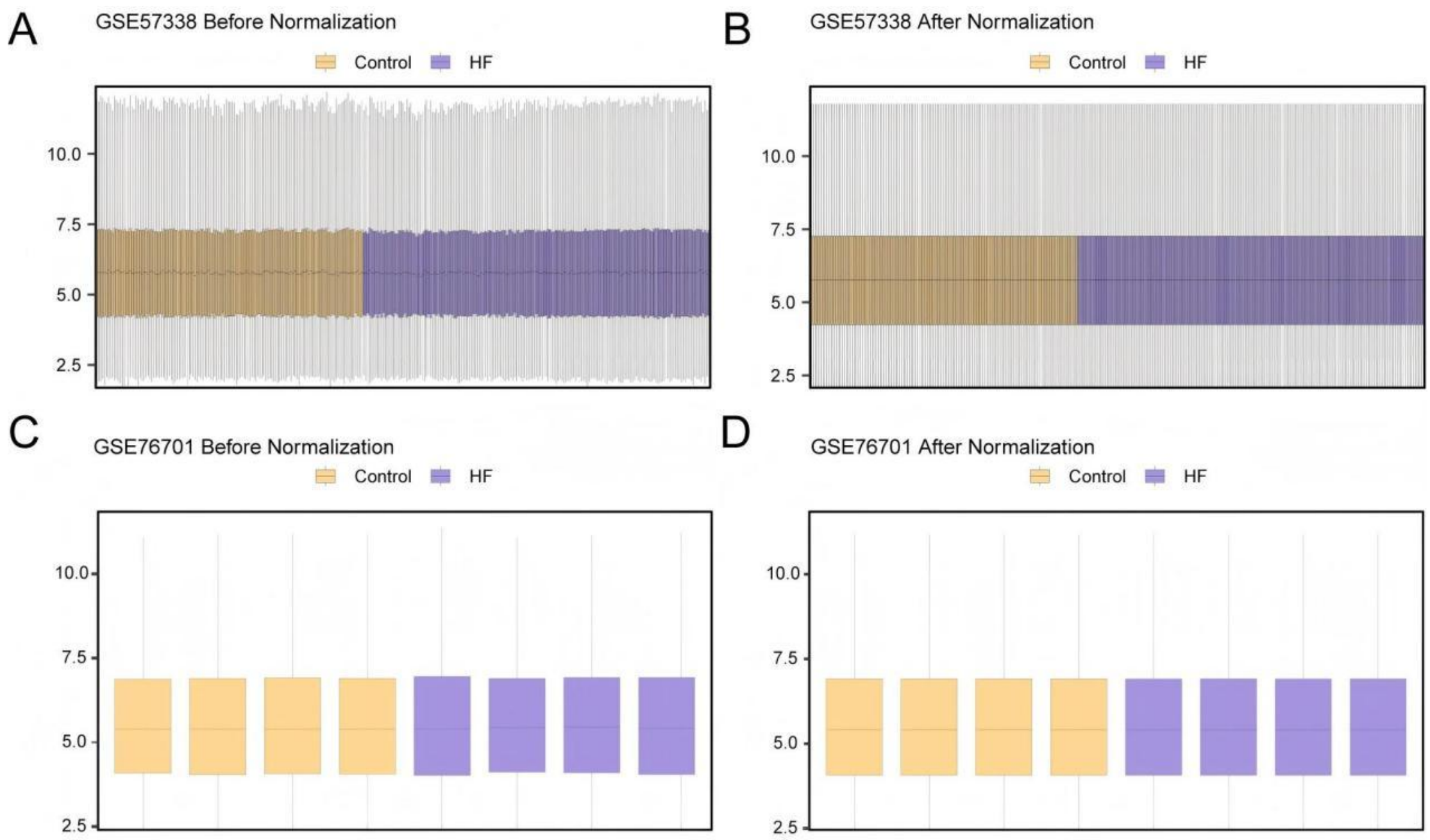

Supplementary Fig. 2 Boxplots of gene expression data before and after normalization. (A–B) GSE57338 before (A) and after (B) normalization. (C–D) GSE76701 before (C) and after (D) normalization.
